# Illusions from the Administration of Chloroform during Menstruation

**Published:** 1865

**Authors:** 


Illusions from the Administation of Chlorofrom during Menstruation.—The *Canada Lancet* says that Dr. Kidd affirms that chloroform when administered by inhalalion, during the period of menstruation, may have the effect of inducing the belief that an assault had been attempted in a criminal way whilst under its influence. Now, although we can not, from our own experience, connect with certainty the fact of menstruation with this effect in more than a single instance, we are cognizant of three well marked cases of the kind occuring in this city, and rumor speaks of several others. We were well acquainted with an elderly gentleman whose wife was so firmly convinced that a dentist had endeavored to take improper liberties with her whilst under the influence of chloroform, that he had much difficulty in convincing her that he, the husband, had not left her side during the whole time.

